# HBQ1 Acts as a Prognostic Biomarker and Tumor Suppressor Linked to Antigen Presentation in Glioma

**DOI:** 10.1002/cbf.70323

**Published:** 2026-10-13

**Authors:** Cheng Xie, Qinglin Zhong, Mingang Zou, Qianliang Huang, Lukui Chen, Wenjin Wei

**Affiliations:** ^1^ Department of Neurosurgery Southern Medical University Hospital of Integrated Traditional Chinese and Western Medicine, Southern Medical University Guangzhou Guangdong Province China; ^2^ Department of Neurosurgery, Ganzhou Hospital‐Nanfang Hospital Southern Medical University (Ganzhou People's Hospital) Ganzhou Jiangxi China

**Keywords:** antigen presentation, GABAergic signal pathway, Glioma, HBQ1, neuron‐glioma synapses

## Abstract

Glioma is a highly aggressive brain tumor associated with substantial morbidity and mortality and with limited treatment options. This study investigates the functional role of Hemoglobin Subunit Theta 1 (HBQ1) in glioma and evaluates its potential as a prognostic biomarker. Using integrated bioinformatics approaches, we demonstrated that HBQ1 expression was significantly downregulated in glioma tissues compared to normal brain tissues and was inversely correlated with tumor grade. Survival analysis indicated that lower HBQ1 expression is associated with poorer overall survival and disease‐free survival. Functional enrichment analyses connected HBQ1 to key pathways including antigen presentation (MHC II), calcium signaling, mitogen‑activated protein kinase (MAPK) signaling, and γ‑aminobutyric acid (GABA)‑ergic synapse. *In vitro*, overexpression of HBQ1 in U87 cells markedly suppressed proliferation, migration, and invasion, while enhancing antigen presentation. Moreover, HBQ1 expression was significantly correlated with important molecular markers such as isocitrate dehydrogenase (IDH) mutation, 1p/19q co‐deletion, and O6‐methylguanine‐DNA methyltransferase (MGMT) promoter methylation, as well as with genes involved in glutamate‐GABA metabolism. Collectively, our findings identify HBQ1 as a novel prognostic biomarker and a potential tumor suppressor in glioma, possibly through modulating the immune microenvironment and neuron–glioma crosstalk.

## Significance

This study addresses the lack of effective prognostic biomarkers for glioma by investigating HBQ1 function. Our findings demonstrate that HBQ1, related to antigen presentation, suppresses glioma cell proliferation, migration, and invasion, providing new insights into glioma tumorigenesis. These results are significant because HBQ1 may serve as a novel prognostic marker, offering guidance for glioma prognostic evaluation and target exploration.

## 1 Introduction

Glioma is recognized as the most common and aggressive primary brain tumor, characterized by rapid cellular proliferation, invasive behavior, and poor prognosis. This malignancy responds inadequately to conventional treatments, including surgical resection, radiotherapy, and temozolomide‐based chemotherapy [1]. Despite recent therapeutic advances, the median survival of glioma patients remains approximately 15 months, with a 5‐year survival rate below 10% [2]. These persistent challenges underscore the need to identify novel molecular targets that may reveal vulnerabilities for therapeutic intervention.

Hemoglobin Subunit Theta 1 (HBQ1) is a protein‐coding gene belonging to the hemoglobin family, known for its role in oxygen transport. During early embryonic development, the HBQ1 protein functions as an oxygen carrier, ensuring adequate oxygen supply to the developing embryo [3]. To date, research on HBQ1 in disease contexts remains limited. Its association with hemoglobinopathies such as thalassemia is established [4]; in these conditions, mutations or dysregulation disrupt hemoglobin synthesis and contribute to anemia [5, 6]. Additionally, some studies suggest that HBQ1 may be implicated in other conditions, such as neurological disorders [7, 8], though this remains poorly characterized. In oncology, HBQ1 has been implicated in tumor progression through pathways related to proliferation [9], chemoresistance [10], and the regulation of immune responses [11]. Moreover, it may participate in hypoxic responses within the tumor microenvironment (TME) [12], highlighting its potential as a diagnostic or prognostic biomarker in cancer.

In this study, we evaluated the expression pattern of HBQ1 in glioma and assessed its correlation with clinical outcomes. Using integrated bioinformatics approaches, we performed a preliminary exploration of HBQ1's functional role in tumor biology. Furthermore, we investigated the effects of HBQ1 overexpression on glioma cell proliferation, migration, and invasion. Our findings underscore the prognostic relevance of HBQ1 in glioma and suggest its potential as a tumor‐suppressive molecule.

## 2 Methodology

### 2.1 Data Acquisition and Bioinformatics Databases

Clinical and RNA‐sequencing data for 33 cancer types were obtained from The Cancer Genome Atlas (TCGA; https://portal.gdc.cancer.gov). RNA‐seq data in Fragments Per Kilobase of transcript per Million mapped reads (FPKM) format were converted to transcripts per million (TPM) for downstream analysis. The Chinese Glioma Genome Atlas (CGGA; https://www.cgga.org.cn) was utilized for differential expression, survival, and correlation analyses. Protein expression and subcellular localization data for HBQ1 were retrieved from the Human Protein Atlas (HPA; https://www.proteinatlas.org) [13]. Genomic alteration frequencies of HBQ1 were assessed using the cBio Cancer Genomics Portal (cBioPortal) (http://www.cbioportal.org) [14] on 1106 TCGA samples, and mutation spectrum analysis was performed via the Somatic Mutations in Cancer (COSMIC) database (https://cancer.sanger.ac.uk/cosmic) [15].

### 2.2 Survival and Prognostic Analysis

Overall survival (OS) and disease‐free survival (DFS) data for glioma patients were obtained from TCGA and CGGA. Kaplan–Meier survival curves were generated, and log‐rank tests were applied to compare groups stratified by the median expression level of HBQ1. Univariate Cox regression was used to evaluate the association between HBQ1 expression and survival outcomes. The predictive accuracy of HBQ1 expression for patient survival at 1, 3, and 5 years was evaluated using time‐dependent receiver operating characteristic (ROC) curve analysis, and the corresponding area under the curve (AUC) values were calculated.

### 2.3 Functional Enrichment Analysis

Differentially expressed genes (DEGs) between groups were identified from TCGA glioma transcriptome data using thresholds of |log_2_ fold change| > 1.0 and adjusted *p* < 0.05. Gene Ontology (GO) and Kyoto Encyclopedia of Genes and Genomes (KEGG) pathway enrichment analyses were performed using the Metascape platform. Gene Set Enrichment Analysis (GSEA) was conducted to identify signaling pathways associated with HBQ1 expression levels.

### 2.4 Cell Culture and Transfection

The human glioblastoma cell line U87 was obtained from the American Type Culture Collection (RRID: CVCL_0022, ATCC) and maintained in Dulbecco's Modified Eagle's Medium (DMEM; Hyclone) supplemented with 10% fetal bovine serum (FBS; Hyclone), at 37°C in a humidified atmosphere containing 5% CO_2_. For HBQ1 overexpression, the coding sequence of HBQ1 [11] was cloned into the pLenti6/V5 vector (RiboBio). Recombinant plasmid DNA was transiently transfected into U87 cells using Lipofectamine 2000 (Thermo Fisher) according to the manufacturer's instructions. Cells were harvested 24–72 h post‐transfection for subsequent assays.

### 2.5 Western Blot Analysis

Cells were lysed in radio‐immunoprecipitation assay (RIPA) buffer containing protease and phosphatase inhibitors on ice. Protein concentration was determined using a bicinchoninic acid (BCA) assay. Equal amounts of protein were separated by sodium dodecyl sulfate‐polyacrylamide gel electrophoresis, transferred to polyvinylidene difluoride (PVDF) membranes (PTG), and blocked with 5% bovine serum albumin. Membranes were incubated overnight at 4°C with primary antibodies against HBQ1 (PTG) and glyceraldehyde‐3‐phosphate dehydrogenase (GAPDH) (CWbio), followed by incubation with horseradish peroxidase (HRP)‐conjugated secondary antibodies. Protein bands were visualized using chemiluminescence reagents. All experiments were independently repeated 3 times.

### 2.6 Cell Proliferation Assays

For the colony formation assay, 1000 cells per well were seeded into six‐well plates and cultured for 14 days. Colonies were fixed with 10% formaldehyde, stained with 0.1% crystal violet, and counted (colonies containing more than 50 cells). For the EdU assay, cell proliferation was measured using the Cell‐Light™ EdU Apollo®567 In Vitro Imaging Kit (RiboBio) according to the manufacturer's protocol. EdU‐positive cells were imaged and quantified under a fluorescence microscope. Both assays were performed in triplicate.

### 2.7 Migration and Invasion Assays

Cell migration and invasion were assessed using Transwell chambers (8‐μm pore size). For migration, 1 × 10^4^ cells in serum‐free Dulbecco's Modified Eagle Medium (DMEM) were seeded into the upper chamber, with DMEM containing 10% fetal bovine serum (FBS) in the lower chamber as a chemoattractant. After 6 h of incubation, cells on the lower surface were fixed, stained, and counted. For the invasion assay, chambers were pre‐coated with Matrigel^TM^, and cells were incubated for 8 h. Experiments were repeated at least three times independently.

### 2.8 Phagocytosis Assay (pHrodo™ Red Staining)

Cell phagocytosis was detected using pHrodo™ Red Staining. All cells were cultured at 37°C under 5% carbon dioxide (CO_2_). U87 cells were maintained in high‐glucose DMEM supplemented with 10% FBS and 1% penicillin‐streptomycin, while macrophages (Mø, RRID: CVCL_0493, ATCC, USA) were cultured in RPMI‐1640 medium supplemented with 10% FBS and antibiotics. For co‐culture assays, U87 cells were seeded at 2 × 10^5^ cells/well and macrophages were seeded at 1.5 × 10^5^ cells/well. U87 cells were labeled with pHrodo™ SE dye following the manufacturer's protocol. HBQ1‐overexpressing U87 cells were co‐cultured with macrophages for 6 h. After fixation and DAPI staining, macrophage phagocytic activity was quantified by counting intracellular pHrodo™ SE‐positive puncta under fluorescence microscopy. U87 cells lacking HBQ1 overexpression served as the control group. Both oe‐HBQ1 and control U87 cells were labeled with pHrodo™ SE dye. All experiments were performed in triplicate.

### 2.9 Statistical Analysis

All data are presented as mean ± standard deviation (SD) from at least three independent experiments. Statistical analyses were performed using SPSS 24.0 (SPSS Inc.) and GraphPad Prism 10.0 (GraphPad Software). Group comparisons were made using Student's *t*‐test or one‐way ANOVA. Categorical variables were compared using Chi‐square or Fisher's exact test. Survival differences were analyzed by the Kaplan–Meier method with log‐rank test. A two‐tailed *p*‐value < 0.05 was considered statistically significant.

## 3 Results

### 3.1 Expression Levels of HBQ1 in Glioma

Pan‐cancer analysis of 33 tumor types using R software (v3.6.4) revealed that HBQ1 mRNA expression was significantly upregulated in 13 cancer types and downregulated in 9 others. Notably, both low‐grade glioma (LGG) and glioblastoma (GBM) showed significant downregulation of HBQ1 compared to normal tissues (Figure 1A; *p* < 0.001). Expression analysis across glioma grades using TCGA and CGGA datasets further confirmed that HBQ1 mRNA levels were inversely correlated with tumor grade (Figure 1B,C; *p* < 0.001).

**FIGURE 1:**
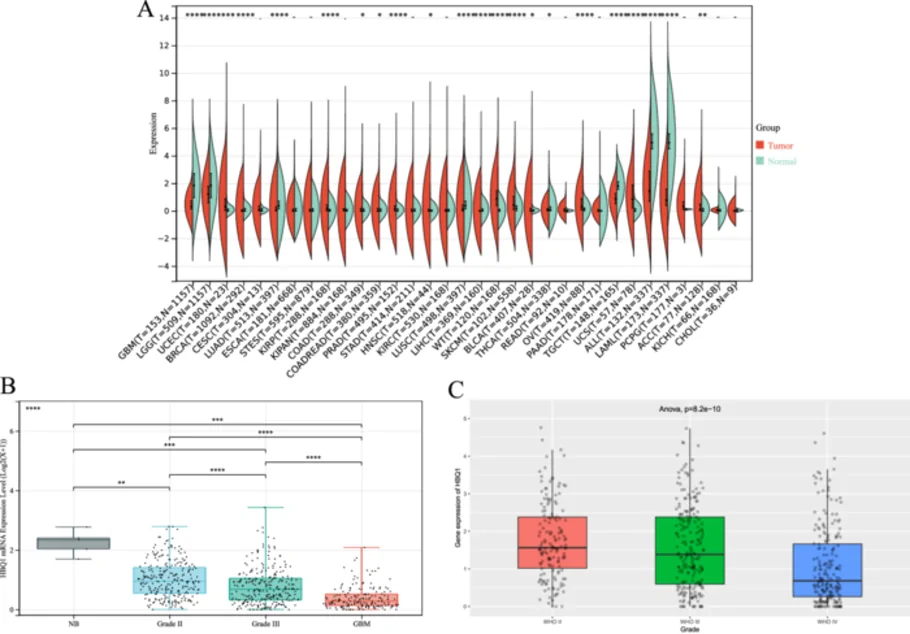
Expression analysis of HBQ1. Expression analysis of HBQ1 between normal and tumor samples in 33 types of tumor (A). Analysis of the expression differences of HBQ1 in different grades of glioma using the Cancer Genome Atlas (TCGA) and Chinese Glioma Genome Atlas (CGGA) databases (B, C).

Analysis of the HPA indicated broad expression of HBQ1 protein across central nervous system cell types, including neurons, microglia, oligodendrocytes, and vascular cells (Figure 2A). Subcellular localization suggested HBQ1 presence in lipid droplets and the cytoplasm (Figure 2B,C). Immunohistochemistry (IHC) data from the HPA demonstrated moderate HBQ1 expression in normal brain tissues, whereas low or undetectable levels were observed in LGG and high‐grade glioma tissues (Figure 2D). These findings suggest that reduced HBQ1 expression may be involved in glioma progression.

**FIGURE 2:**
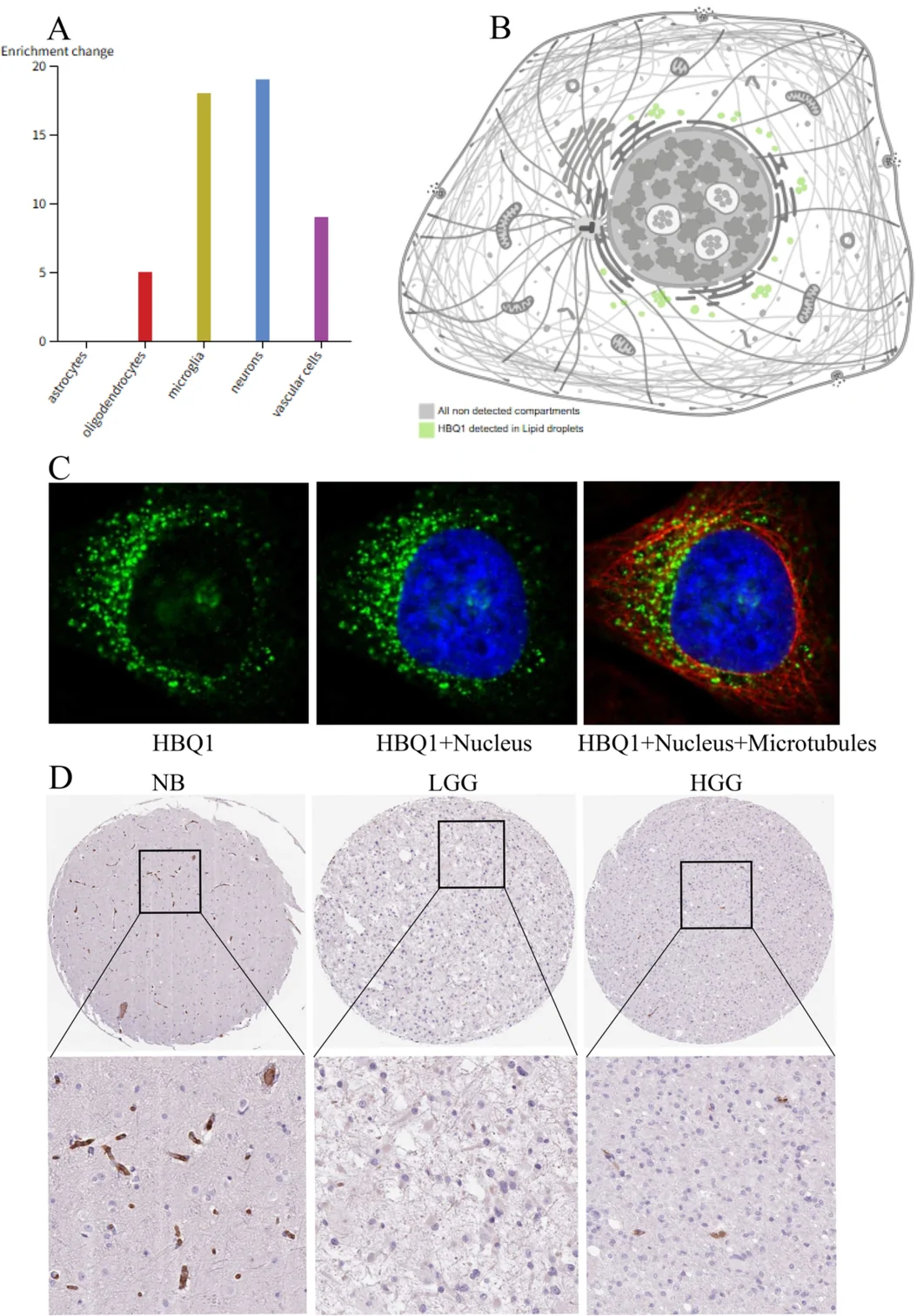
Distribution of HBQ1 protein and its expression status in normal and tumor tissues. The expression of HBQ1 protein in different cells of the central nervous system (A). Cellular localization of HBQ1 protein (B, C). Expression level of HBQ1 protein in normal and different grades of tumor tissues through IHC (D). Images were obtained from Human Protein Atlas (HPA).

### 3.2 Prognostic Significance of HBQ1 in Glioma

We further evaluated the correlation between HBQ1 expression and key molecular markers. The univariate analysis revealed that higher HBQ1 expression levels in gliomas were significantly associated with isocitrate dehydrogenase (IDH) mutations (Figure 3A, *p* < 0.001), 1p/19q co‐deletion status (Figure3B, *p* < 0.001), and O6‐methyguanine‐DNA methyltransferase (MGMT) promoter methylation (Figure 3C, *p* < 0.05). Survival analysis based on TCGA and CGGA data showed that high HBQ1 expression was associated with significantly better OS (Figure 3D,E, *p* < 0.001) and DFS (Figure 3F, *p* = 4.5 × 10^−10^). Time‐dependent ROC analysis indicated that HBQ1 expression had substantial predictive value for patient survival at 1, 3, and 5 years, with AUC values of 0.78, 0.83, and 0.83, respectively (Figure 3G).

**FIGURE 3:**
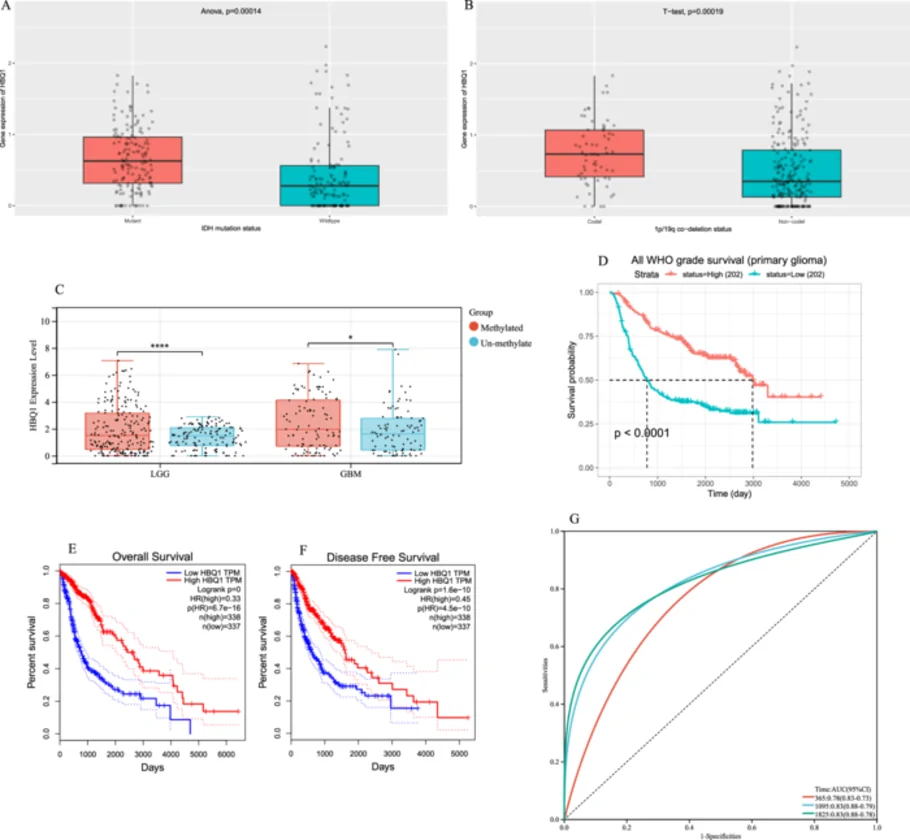
Prognostic and survival analysis of HBQ1 in glioma. Correlation analysis of HBQ1 expression level with IDH‐mutant, 1P/19q co‐deletion and MGMT methylation status by using the CGGA database (A–C). The Kaplan–Meier (K–M) survival analysis illustrated the relationship between HBQ1 expression and overall survival (OS) and disease‑free survival (DFS) (D–F). The receiver operating characteristic (ROC) curve was used to evaluate the discriminative ability of HBQ1 for predicting patient prognosis (G).

### 3.3 Mutation Landscape of HBQ1 in Glioma

Analysis of 1106 glioma samples from the TCGA Pan‐Cancer Atlas via cBioPortal showed a low somatic mutation frequency of HBQ1 (0.4%; 4/1106), suggesting that HBQ1 mutations are rare and unlikely to be a major prognostic factor in glioma (Figures 4A,B). Further characterization using COSMIC revealed that missense mutations accounted for 56.34% of alterations, while synonymous mutations comprised 30.99% (Figure 4C). The most common substitution types were G > A (32.79%), C > T (29.51%), and A > G (16.39%) transitions (Figure 4D).

**FIGURE 4:**
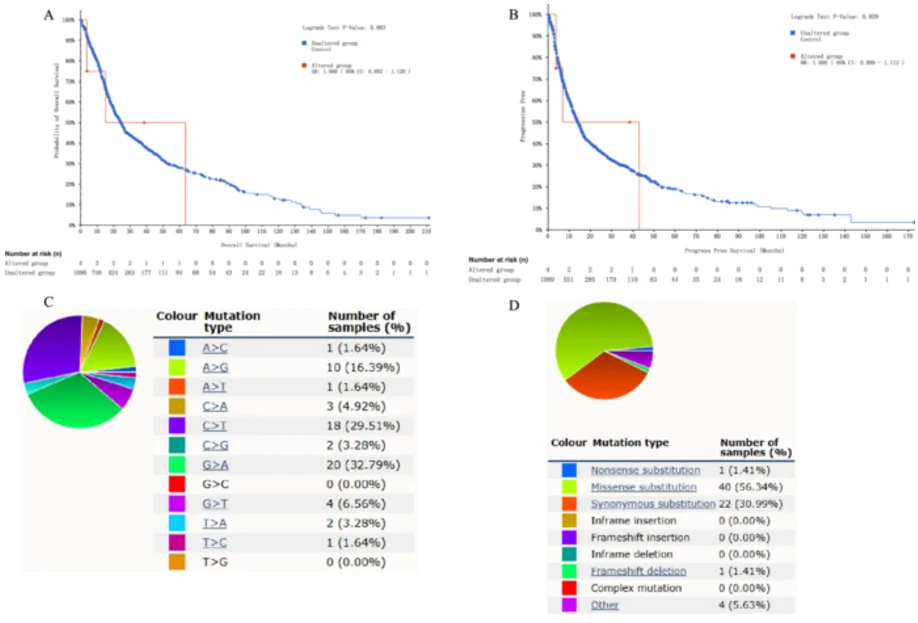
Mutations of HBQ1 in Glioma. HBQ1 mutations and glioma patient prognosis from the cBio Cancer Genomics Portal (cBioPortal) (A, B). The types of HBQ1 mutations were also assessed using the Somatic Mutations in Cancer database (COSMIC) (C, D).

### 3.4 Functional Enrichment of HBQ1‐Associated Genes

A total of 2541 DEGs were identified (|log_2_FC | > 1.0, FDR < 0.05), including 1295 upregulated and 1246 downregulated genes (Figure 5A). GO enrichment analysis highlighted biological processes such as humoral immune response, lymphocyte‐mediated immunity, and adaptive immune response. Cellular component terms included synapse, GABAergic synapse, and MHC class II protein complex. Molecular functions were enriched for antigen binding, MHC class II receptor activity, and glutamate‐gated calcium channel activity (Figure 5B). KEGG pathway analysis revealed involvement in p53 signaling, calcium signaling, PI3K‐Akt signaling, T cell receptor signaling, GABAergic synapse, and antigen processing and presentation (Figure 5C). GSEA further revealed that HBQ1 was negatively correlated with calcium signaling, MAPK signaling, oxidative phosphorylation, Wnt signaling, and GnRH signaling. In contrast, it was positively correlated with multiple pathways, including p53 signaling, apoptosis, and immune‐related pathways (such as antigen presentation and natural killer cell‐mediated cytotoxicity) (Figure 5D,E).

**FIGURE 5:**
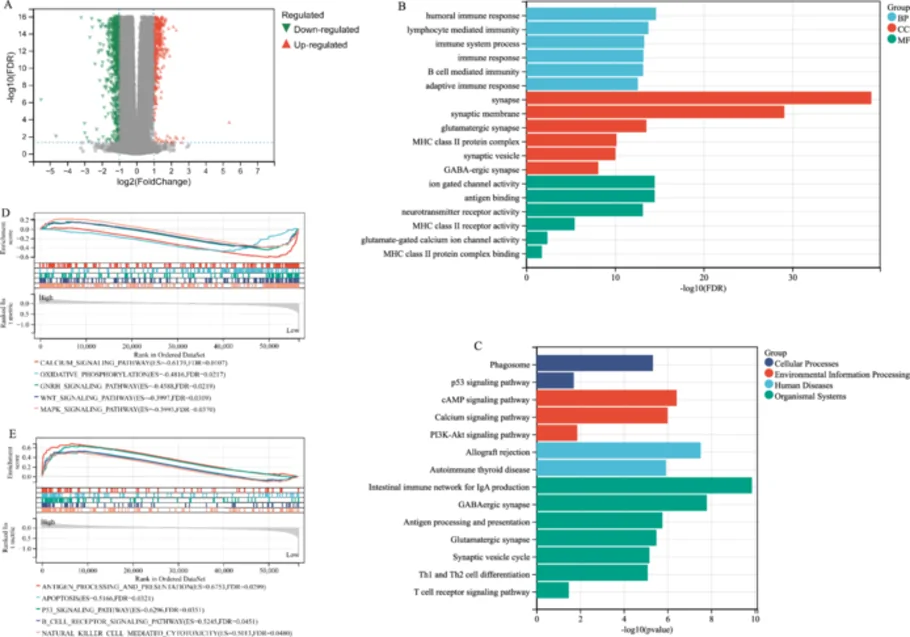
Functional enrichment analysis. Differential analysis of genes related to HBQ1 (A). Gene Ontology (GO) (B), Kyoto Encyclopedia of Genes and Genomes (KEGG) (C), and Gene Set Enrichment Analysis (GSEA) (D, E) analysis of differentially expressed genes (DEGs).

### 3.5 HBQ1 Overexpression Suppresses Glioma Cell Malignant Phenotypes

To investigate the functional role of HBQ1, we constructed an overexpression plasmid (oe‐HBQ1). Transfection into U87 cells confirmed successful HBQ1 upregulation at the protein level (Figure 6A). Overexpression of HBQ1 significantly inhibited cell proliferation, as shown by colony formation (Figure 6B,C) and EdU incorporation assays (Figure 6D,E). Transwell migration assays indicated reduced migratory ability in HBQ1‐overexpressing cells (Figure 6F,G), and Matrigel‐based invasion (Boyden) assays demonstrated impaired invasive capacity (Figure 6H,I). Together, these results suggest that HBQ1 acts as a tumor suppressor in glioma cells by inhibiting proliferation, migration, and invasion.

**FIGURE 6:**
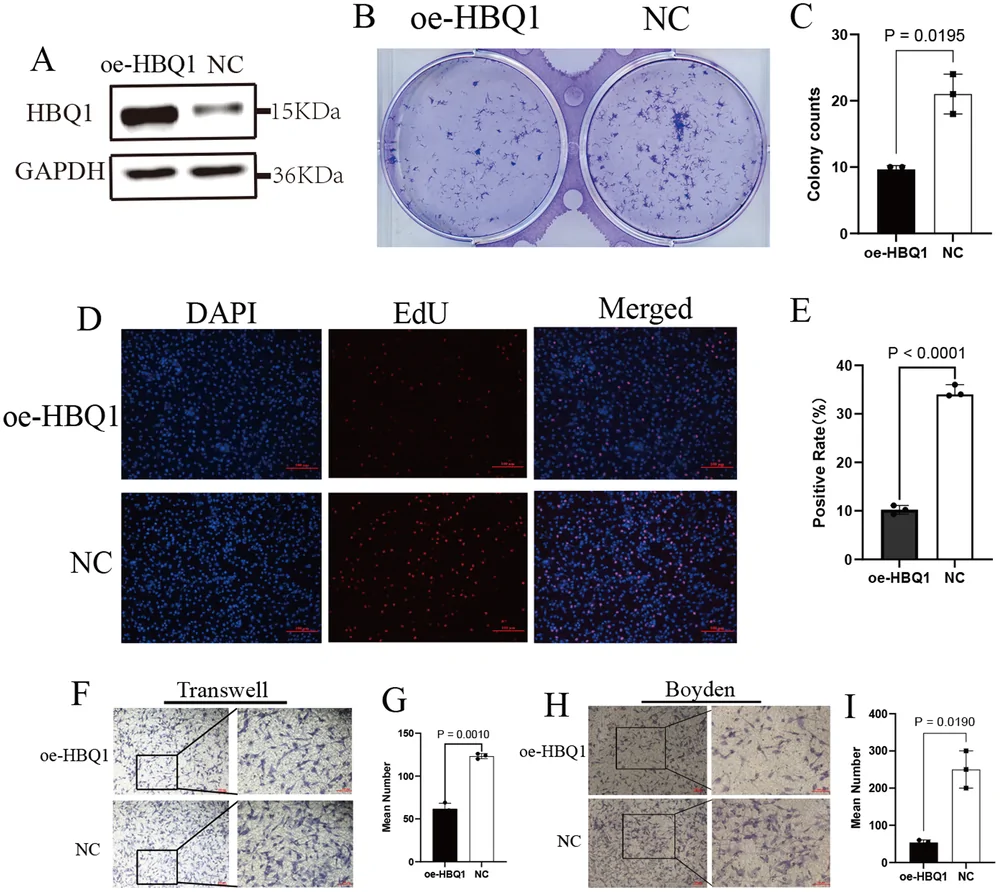
Verification of the functional effects of HBQ1 overexpression in glioma cells. Western blot was used to verify the expression of HBQ1 (A). Overexpressed HBQ1 could significantly inhibit the proliferation of U87 cell through Colony‐formation assays (B, C) and EdU (D, E). Transwell (F, G) and Boyden (H, I) assays showed that up‐regulated HBQ1 could restrain the migration and invasion of glioma.

### 3.6 HBQ1 Overexpression Enhances Macrophage‐Mediated Uptake of Tumor Cells

Our GO and GSEA analyses revealed strong enrichment of HBQ1‐associated genes in MHC class II‐related functions and antigen presentation, suggesting that HBQ1 might be involved in the antigen presentation process within the glioma microenvironment. Therefore, we co‐cultured macrophages with U87 tumor cells overexpressing HBQ1 to further investigate the effect of HBQ1 on tumor cell phagocytosis. The overexpression of HBQ1 enhanced macrophage‐mediated tumor cell uptake (Figure 7), which preliminarily indicates that HBQ1 overexpression renders glioma cells more susceptible to macrophage phagocytosis in the TME.

**FIGURE 7:**
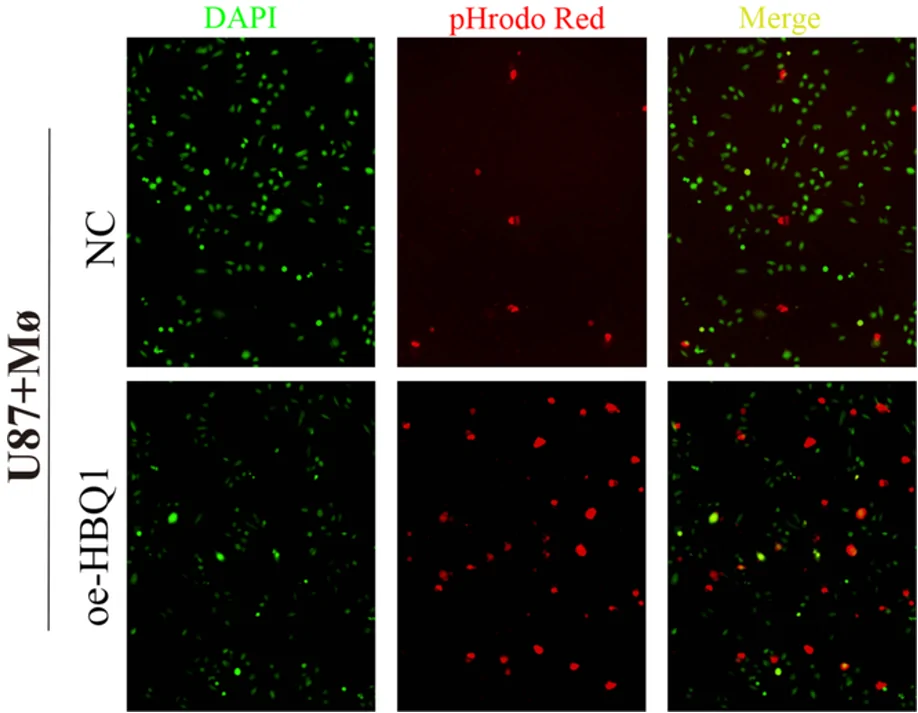
Detection of HBQ1‑induced macrophage‑mediated phagocytosis of tumor cells. The pHrodo™ Red staining experiment demonstrated that HBQ1‐overexpressing (oe‐HBQ1) cells were more susceptible to phagocytosis than controls. MØ, macrophages. Nuclei were stained with 4'6‑diamidino‑2‑phenylindole (DAPI). NC, negative control; oe‑HBQ1, HBQ1 overexpression; U87+Mo, U87 glioma cells co‑cultured with macrophages.

## 4 Discussion

Glioma, the most prevalent and aggressive primary brain tumor, originates from glial cells and is characterized by pronounced molecular and genetic heterogeneity. This complexity presents major therapeutic challenges and underscores the urgent need to elucidate the mechanisms driving tumor progression and treatment resistance [16, 17]. In this study, we investigated the expression pattern and clinical significance of HBQ1 in glioma.

We found that HBQ1 expression is significantly down‐regulated in tumor compared to normal brain tissue and is inversely correlated with tumor grade. Importantly, elevated HBQ1 expression was associated with better overall and disease‐free survival, identifying HBQ1 as a potential prognostic biomarker in glioma. These observations provide new insights into glioma biology and suggest that HBQ1 may serve as a novel prognostic biomarker and a potential therapeutic candidate.

IDH1 mutation, 1p/19q codeletion, and MGMT promoter methylation are well‐established molecular markers that define glioma subtypes and predict therapeutic response [18]. IDH1 mutations, common in LGG, lead to 2‐hydroxyglutarate accumulation and a hypermethylated phenotype (G‐CIMP) linked to better prognosis [19, 20]. The 1p/19q co‐deletion, characteristic of oligodendrogliomas, synergizes with IDH1 mutation and predicts sensitivity to alkylating chemotherapy [21, 22, 23]. MGMT promoter methylation enhances temozolomide efficacy by impairing DNA repair [24]. In our analysis, higher HBQ1 expression correlated positively with the presence of IDH1 mutations, 1p/19q co‐deletion, and MGMT methylation. This association suggests that HBQ1 may be involved in modulating chemotherapy response, positioning it as a potential biomarker for treatment sensitivity. However, the exact mechanistic role of HBQ1 in this context requires further investigation.

Immunotherapy represents a promising avenue for glioma treatment, but its efficacy is limited by immune evasion mechanisms, including downregulation of antigen presentation and immunosuppressive factor production [25, 26]. Somatic mutations in antigen‐presenting genes such as HLA and B2M may further promote immune escape [27]. Therefore, strategies that restore or enhance antigen presentation within the TME could improve immunotherapy outcomes. Recent studies have also demonstrated that HBQ1 can serve as a potential target for immunotherapy in diseases [11, 12, 28]. These observations suggest that HBQ1 may exert broader immunoregulatory functions across cancer types. Consistent with this hypothesis, our study indicates that HBQ1 may be a novel modulator of antigen presentation in glioma. Transcriptomic profiling revealed that HBQ1‐associated genes were significantly enriched in MHC class II‐related pathways and pathways involved in antigen processing and presentation. GSEA analysis further showed that HBQ1 expression was positively correlated with immune‐related pathways. Functional validation demonstrated that overexpression of HBQ1 in U87 glioma cells markedly enhanced macrophage‐mediated phagocytosis, suggesting that HBQ1 could potentiate antigen presentation within the TME. Collectively, these findings position HBQ1 as a promising candidate for overcoming antigen‐presentation deficits and boosting the efficacy of immunotherapies in glioma. However, the underlying molecular mechanisms—particularly how HBQ1 interacts with MHC class II machinery—remained to be elucidated. Future studies using in vivo glioma models, proteomic interaction screens, and combination with immune checkpoint inhibitors are warranted to establish HBQ1 as a viable therapeutic target.

The TME, particularly interactions between glioma cells and neurons, plays a critical role in tumor progression [29, 30, 31]. Neuron‐glioma synapses (NGSs) facilitate pro‐tumor signaling through ion channels, neurotransmitters, and gap junctions [32, 33, 34]. Glutamate released from neurons activates calcium influx and mitogen‑activated protein kinase (MAPK) signaling in glioma cells, promoting growth and invasion [35]. Our GSEA indicated that low HBQ1 expression is associated with activation of MAPK and calcium signaling pathways, implying that HBQ1 may suppress glioma progression by inhibiting these pathways and disrupting NGS function.

Furthermore, glutamate can attenuate GABA‐mediated inhibition at NGSs by downregulating expression of potassium‑chloride cotransporter‑2 (KCC2, SLC12A5) or upregulating expression of sodium‑potassium‑chloride cotransporter‑1 (NKCC1, SLC12A2) [36]. Gamma‑aminobutyric acid type A receptor subunit alpha1 (GABA) exerts inhibitory effects via chloride influx through gamma‑aminobutyric acid type A receptor (GABAA) receptors such as GABRA1 [37]. Our correlation analysis showed that HBQ1 expression positively correlated with KCC2 and GABRA1, and negatively correlated with NKCC1 (Figure 8A). HBQ1 also correlated negatively with glutamate synthase (GLS) and positively with glutamate dehydrogenase 1 (GLUD1) and glutamate decarboxylase 1 (GAD1) (Figure 8B). These findings suggest that HBQ1 may reduce glutamate availability and promote GABAergic signaling, thereby attenuating excitatory drive in the glioma microenvironment.

**FIGURE 8:**
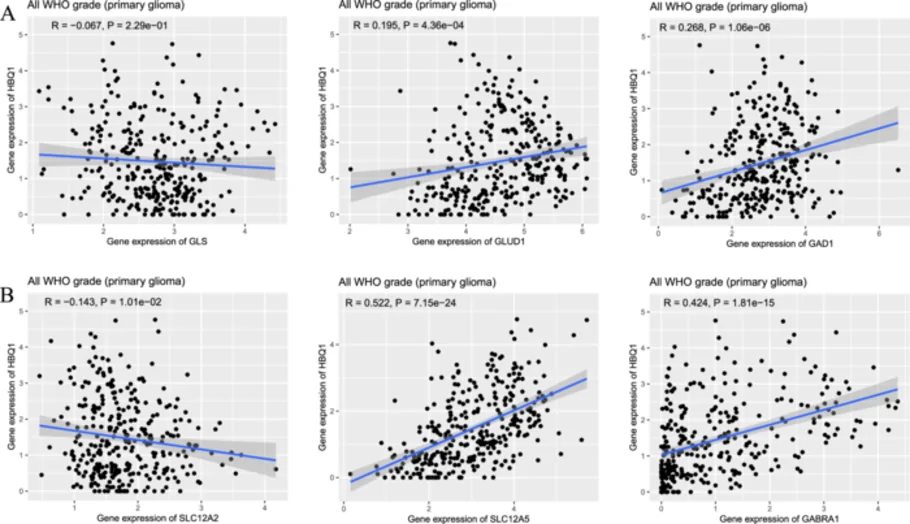
Correlation analysis between HBQ1 and the GABAergic‐related genes. Correlation analysis between HBQ1 and the GABAergic‐related genes was performed using the CGGA database (A and B). R, correlation coefficient.

This study provides preliminary evidence from bioinformatics and cellular experiments supporting a tumor‐suppressive role for HBQ1 in glioma. However, more comprehensive in vitro and in vivo studies are needed to validate its biological functions. The involvement of HBQ1 in antigen presentation and neuron–glioma crosstalk warrants deeper mechanistic exploration to fully understand its therapeutic potential.

## 5 Conclusion

In conclusion, our study identifies HBQ1 as a promising prognostic biomarker in glioma, with potential value for molecular stratification. Elevated HBQ1 expression inhibits glioma cell proliferation, migration, and invasion, suggesting a tumor‐suppressive function. Furthermore, HBQ1 may enhance antigen presentation within the TME and promote GABAergic signaling in neuron–tumor interactions. These findings illuminate the multifaceted role of HBQ1 in glioma pathogenesis and provide a rationale for developing HBQ1‐targeted strategies to improve therapeutic precision and patient outcomes.

## Conflicts of Interest

The authors declare no conflicts of interest.

## Data Availability

Experimental data generated during the current study are available from the corresponding author upon reasonable request.
